# Efficiency of Neurologist-Led Focused Cardiac Ultrasound in the Acute Stroke Pathway (S-FoCUS)

**DOI:** 10.3390/diagnostics16101491

**Published:** 2026-05-14

**Authors:** Eduardo Mariño, Lara Pulido Fraiz, Carlos Hervás-Testal, Ricardo Rigual, Gerardo Ruiz-Ares, Laura Casado, Blanca Fuentes, Esther Pérez-David, Gabriela Guzmán-Martínez, María Alonso de Leciñana, Jorge Rodríguez-Pardo

**Affiliations:** 1 Department of Neurology , Stroke Center, La Paz University Hospital, Universidad Autónoma de Madrid, La Paz University Hospital Research Institute (IdiPAZ), 28046 Madrid, Spain; eduardo.trillo@salud.madrid.org (E.M.); larapulidofraiz@gmail.com (L.P.F.); carlosherv93@hotmail.com (C.H.-T.); ricardojaime.rigual@salud.madrid.org (R.R.); gerardo.ruiz@salud.madrid.org (G.R.-A.); laura.casado@salud.madrid.org (L.C.); blanca.fuentes@salud.madrid.org (B.F.); 2 Cardiac Image Unit, Department of Cardiology, La Paz University Hospital, Universidad Autónoma de Madrid, La Paz University Hospital Research Institute (IdiPAZ), 28046 Madrid, Spain; eperezdavid@gmail.com (E.P.-D.); gabriela.guzman@salud.madrid.org (G.G.-M.)

**Keywords:** cardioembolic stroke, focused cardiac ultrasound, FoCUS, stroke pathway, healthcare efficiency

## Abstract

**Background/Objectives**: Although comprehensive transthoracic echocardiography (TTE) is part of the diagnostic workup in acute ischemic stroke, it is not cost-effective to use it for all patients. Guidelines recommend using it only for selected patients to guide secondary prevention. Neurologist-led, stroke-focused cardiac ultrasound (S-FoCUS) is an emerging bedside screening tool that optimizes cardiac evaluation in acute stroke care. We hypothesize that the implementation of S-FoCUS screening may reduce resource utilization in terms of hospital stay and TTE procedures. **Methods**: We conducted a retrospective before-and-after cohort study of patients with suspected acute ischemic stroke or TIA admitted to our comprehensive stroke center. We compared two 6-month periods: the pre-S-FoCUS period, during which patients underwent TTE as the initial cardiac imaging modality; and the post-S-FoCUS period, during which patients initially underwent S-FoCUS, with subsequent TTE performed only in the presence of abnormal findings or at the clinician’s discretion. We compared the time from admission to first cardiac ultrasound assessment, length of stay, relative reduction in TTE procedures and estimated in-hospital costs. **Results**: The pre-S-FoCUS period included 224 patients, and the post-S-FoCUS period included 229 patients. The S-FoCUS protocol reduced the median time to first cardiac ultrasound assessment by two days (median [IQR] 3 [2–5] vs. 1 [1–2], *p* < 0.001) and the median length of stay by one day (6 [4–9] vs. 5 [3–10], *p* = 0.014). Implementing the S-FoCUS protocol was associated with an exploratory estimate of lower in-hospital costs (approximately €716 per screened patient) driven mainly by a shorter length of stay. The distribution of the frequency of predefined cardioembolic sources was similar between both periods. There was a high agreement rate between the S-FoCUS and TTE findings in patients who underwent both tests: mitral stenosis (κ = 0.78), left atrial severe enlargement (κ = 0.74), left ventricular hypokinesia/akinesia (κ = 0.84), and depressed LVEF (κ = 0.88). **Conclusions**: Neurologist-led S-FoCUS is a feasible triage strategy to improve efficiency in the acute stroke pathway.

## 1. Introduction

Acute ischemic stroke remains one of the leading causes of death and disability worldwide, despite advances in prevention and treatment [1]. Cardioembolic mechanisms account for approximately one-fifth to one-third of all ischemic strokes [2]. Compared with other etiologic subtypes, cardioembolic strokes are associated with earlier recurrence and poorer functional outcomes [3].

In patients with ischemic stroke or transient ischemic attack (TIA), atrial fibrillation is the leading cardioembolic source [4]; thus electrocardiographic monitoring is mandatory. Standard transthoracic echocardiography (TTE) has traditionally been part of first-line structural cardiac assessment. However, due to its limited impact on management in unselected cohorts, the 2018 AHA/ASA guidelines recommended against routine use [5]. The 2019 update and the 2021 prevention guideline endorsed a more selective, management-driven approach [5,6], in line with contemporary analyses demonstrating only modest actionable yield from routine TTE [7]. In routine practice, the delay to echocardiographic assessment varies greatly across systems, typically amounting to a median of two days in tertiary centers [8], but often exceeding this timeframe in smaller or resource-limited hospitals [9]. Furthermore, if TTE is arranged after discharge, the median delay is 30 days [10], which can further impede etiological clarification.

Stroke-focused cardiac ultrasound (S-FoCUS) has emerged as a complementary tool to expedite the initial cardiac assessment in acute stroke care. S-FoCUS is a targeted bedside echocardiographic examination that includes B-mode and color Doppler imaging. It is performed by trained neurologists to address specific clinical questions relevant to stroke diagnosis. It can be performed within minutes inside the stroke unit, potentially reducing the time to the first cardiac assessment [11]. Pilot studies have demonstrated the feasibility of neurologist-led S-FoCUS and have reported good concordance with cardiologist echocardiography for clinically significant findings [12]. More recently, the multicenter S-FoCUS study validated its diagnostic reliability, reporting high sensitivity and specificity, as well as an overall agreement of 0.64 with standard TTE [13]. Training frameworks have also been formalized, with national and European curricula supporting structured implementation under adequate supervision [11,14].

In our healthcare setting, TTE is commonly requested early in the course of hospitalization for ischemic stroke or TIA as part of the diagnostic work up, before definitive etiologic classification is established.

Our aim is to evaluate whether implementing an S-FoCUS-based triage protocol can reduce hospital stay and resource utilization in patients with suspected acute stroke.

## 2. Materials and Methods

### 2.1. Study Design and Setting

We conducted a retrospective observational cohort study at a comprehensive stroke center in Madrid. The study compared two six-month periods: one before and one after the implementation of a neurologist-led S-FoCUS protocol. We selected the same calendar months in both years to improve seasonal comparability and minimize potential differences related to holidays, admission patterns, and staffing fluctuations. During the pre-implementation period (December 2023–May 2024), patients underwent a comprehensive transthoracic echocardiogram (TTE) as the initial examination. During the post-implementation period (December 2024–May 2025), an S-FoCUS examination was performed by a stroke neurologist. In cases of abnormal findings, or whenever was considered necessary by the treating physician, a TTE was subsequently performed.

TTEs were performed in the Unit of Cardiac Imaging for examination by expert cardiologists. For this purpose, patients were transferred from the stroke unit or the neurology ward. S-FoCUS examination was performed at the bedside in the stroke unit and the neurology ward by a board-certified stroke neurologist who was accredited in neurosonology and FoCUS, according to national and European standards [11,14]. Eventually, S-FoCUS could also be performed in the emergency department or in TIA clinics upon admission.

We aimed to evaluate the efficiency of the diagnostic pathway rather than the indications of the cardiac evaluation. Decisions regarding cardiac evaluation were made by the treating stroke team in accordance with routine clinical practice. During the post-implementation period, S-FoCUS was intended to be the default first-line bedside cardiac assessment. However, patients in whom TTE was warranted upfront, S-FoCUS was not usually performed. Potential indications for direct TTE evaluation were analyzed between the periods.

The Ethics Committee of La Paz University Hospital approved this study (code number PI-2025.816). Informed consent was waived due to the retrospective, observational nature of the study.

### 2.2. S-FoCUS Protocol

The S-FoCUS protocol was designed to identify two groups of clinically actionable findings:

(1) Abnormalities potentially relevant to stroke etiologic assessment (cardioembolic sources, indirect markers or relevant valve disease) as follows:

- Depressed left ventricular ejection fraction (LVEF) and regional wall motion abnormalities, by visual estimation.
- Left atrial enlargement, by visual estimation.
- Aortic or mitral stenosis, by B-mode and color Doppler visual estimation. Mitral calcification.
- Aortic arch complex plaques (≥4 mm, mobile, or ulcerated).
- Masses, thrombi, valvular nodules, or vegetations.

(2) Other findings not related to stroke etiology but that might warrant further evaluation as follows:

- Severe left ventricular hypertrophy or left ventricular dilation with preserved LVEF.
- Severe aortic or mitral regurgitation.
- Inferior vena cava dilation.

All findings were recorded as binary (present or absent) or in predefined ordinal categories (e.g., left atrial size: normal, mild, moderate, or severe; left ventricular systolic function: normal, mildly reduced, moderately reduced, or severely reduced). S-FoCUS does not attempt to quantitatively measure chamber areas or volumes, gradients, or valve stenosis/regurgitation severity. In patients with TTE, this was considered the gold standard. Since patent foramen ovale screening was performed using transcranial Doppler, this finding was not included in S-FoCUS.

S-FoCUS examinations were considered abnormal when they showed one or more predefined findings explained in the protocol. Patients with abnormal findings, or significant changes from previous studies, were referred for TTE. Patients with mechanical valves or other conditions requiring a more comprehensive evaluation were examined directly with TTE. Cases in which basic findings could not be determined due to absence of acoustic window were reported as not assessable.

### 2.3. Training and Quality Assurance

All S-FoCUS examinations were performed by a single neurologist who specialized in stroke medicine and had more than eight years of experience in neurosonology. S-FoCUS training followed the national consensus curriculum and consisted of a 26 h theoretical course and practical training conducted in accredited stroke units in accordance with SEC-SEN standards for clinical practitioners [14]. During this proficiency phase, the operator completed over 100 supervised examinations, each of which was validated by a senior cardiologist to ensure diagnostic reliability.

### 2.4. Population

We included all patients admitted to the Department of Neurology with suspected acute ischemic stroke or TIA during the two study periods who underwent cardiac ultrasound examinations (either TTE or S-FoCUS) as part of the diagnostic workup. Stroke mimics were retained in the main analysis in case the cardiac ultrasound was performed before an alternative diagnosis was established (i.e., clinical suspicion remained ischemic stroke or TIA at the moment of cardiac evaluation).

There were no predefined exclusion criteria; patients who did not receive a cardiac ultrasound were excluded. This may have included critically ill patients, patients with recent cardiac imaging, or patients for whom cardiac imaging was deemed unnecessary based on clinical criteria.

### 2.5. Variables and Outcomes

Clinical variables included age, sex, and stroke severity measured by NIHSS score at admission and final diagnosis after complete diagnostic work-up according to the Spanish Society of Neurology etiological classification criteria [15]. Efficiency variables comprised time from hospital admission to the first cardiac ultrasound assessment (TTE in the pre-S-FoCUS period and S-FoCUS and/or TTE in the post-S-FoCUS period), in-hospital length of stay (LOS), and the proportion of patients undergoing TTE. Additionally, we performed an exploratory estimate of in-hospital cost differences using institutional analytical accounting data based on the All-Patient Refined-DRG (APR-DRG) classification [16].

Performance evaluation variables included the frequency of predefined high-risk cardiac abnormalities (as defined in the S-FoCUS protocol) and the agreement between S-FoCUS and TTE when performed on the same patient.

Study variables were extracted from patients’ electronic records, pseudonymized, and collected and managed using Research Electronic Data Capture (REDCap) [17], which is hosted on the secure IdiPAZ server.

### 2.6. Statistical Analysis

Continuous variables were summarized as means ± standard deviation (SD) or medians with interquartile ranges (IQRs), and categorical variables were summarized as frequencies and percentages. Due to the skewed distribution of in-hospital LOS, medians were reported for clinical outcomes, while means were used for economic estimations to allow for linear monetary transformation into institutional costs.

We performed inter-period comparisons using the Mann–Whitney U test for continuous variables and the chi-square or Fisher’s exact tests for categorical data. Multivariable linear regression was used to identify independent predictors of LOS and specifically assess the impact of S-FoCUS performance. NIHSS at admission was included in the model as a covariate to adjust for stroke severity. Sensitivity analyses were performed including only patients with ischemic stroke or TIA. An additional sensitivity analysis compared patients without a predefined indication for direct TTE.

The economic impact was estimated from a hospital perspective and focused primarily on inpatient stay costs. Savings were calculated by multiplying the mean LOS difference by the weighted mean daily inpatient cost.

Agreement between S-FoCUS and TTE for pre-specified cardioembolic findings was assessed using Cohen’s kappa (κ) statistic: kappa values ≥ 0.60 indicate good agreement. The 95% confidence intervals (CIs) for the kappa coefficients were calculated using a nonparametric bootstrap method with 2000 resampling iterations.

All tests were two-sided, and *p* < 0.05 was considered statistically significant. Analyses were conducted using IBM SPSS Statistics (version 25.0, Armonk, NY, USA).

## 3. Results

### 3.1. Baseline Characteristics

**Figure 1:**
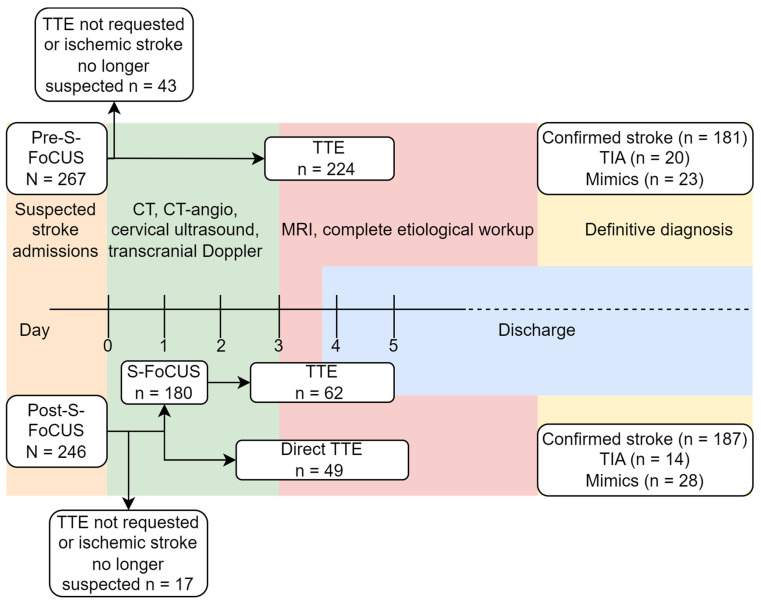
Study flowchart.

A total of 513 patients were admitted with suspected ischemic stroke or TIA during the study periods: 267 in the pre-S-FoCUS period and 246 in the post-S-FoCUS period. In the pre-S-FoCUS cohort, 224 patients (83.9%) underwent cardiac imaging as part of the diagnostic workup of ischemic stroke and were included in the study. In the post S-FoCUS cohort, 229 (93.1%) underwent the corresponding imaging assessment and were included (Figure 1).

Clinical characteristics and final diagnoses are summarized in Table 1. No significant differences were found between the two study periods. Reasons for direct referral to TTE and for escalation from S-FoCUS to TTE are summarized in Supplementary Table S1 and Table S2, respectively.

### 3.2. Pathway Efficiency

Regarding resource utilization, 180 patients (78.6%) in the post-S-FoCUS cohort were initially screened with S-FoCUS, while 49 (21.4%) underwent direct TTE. Among those screened with S-FoCUS, 62 (34.4%) were subsequently referred for standard TTE. In absolute terms, the number of TTE examinations decreased from 224 in the pre-implementation period to 111 in the post-implementation period. Considering all admissions, this corresponded to a reduction from 83.9 (224/267) to 45.14% (111/246). Data comparing efficiency outcomes between the two study periods and patients screened with S-FoCUS are shown in Table 2. In the post-S-FoCUS period, the median time from admission to the first cardiac ultrasound assessment decreased by two days. The proportion of patients imaged within 24 h increased from 5% to 64%, and the median in-hospital stay declined by one day in the overall cohort. This reduction was driven by patients managed with S-FoCUS, with no differences in patients directly examined using TTE. In sensitivity analyses, the reduction in LOS was maintained in the subgroup of patients with confirmed ischemic stroke and TIA (Supplementary Tables S3 and S4), and also in the subgroup with no potential indications for direct TTE (Supplementary Table S5).

We found no delay in time from admission to TTE in patients screened with S-FoCUS compared to the pre-S-FoCUS cohort (median 4 days [IQR 1–5] vs. 3 days [IQR 2–5], *p* = 0.435).

Univariate analyses showed a significant correlation between NIHSS and LOS (r = 0.230, *p* < 0.001). In multivariable analysis adjusted for NIHSS and final diagnosis, the post-S-FoCUS period was not associated with a statistically significant reduction in LOS (β = −0.74 days, 95% CI −2.35 to 0.87; *p* = 0.367). However, patients managed with S-FoCUS only showed a significant reduction in adjusted LOS (β = −2.40 days, 95% CI −3.81 to −0.99; *p* = 0.001) (Table 3). We investigated whether the clinical characteristics of the S-FoCUS-only group differed from those in the pre-S-FoCUS cohort, finding no differences in NIHSS distribution [mean 5.50 vs. 5.85, median 3 (1–8) vs. 3 (1–9), *p* = 0.600] or proportion of confirmed ischemic stroke (78% vs. 81%, *p* = 0.506).

To estimate the savings achieved by S-FoCUS implementation, we calculated a weighted mean cost per stay of €592 per day, based on data from the hospital’s analytical accounting systems. The observed mean LOS difference between the pre- and post-implementation cohorts (8.85 ± 8.21 vs. 8.25 ± 9.36 days) corresponded to an exploratory estimated difference of approximately €355.2 per patient. Among patients initially screened with S-FoCUS, the mean LOS reduction was 1.21 days (8.85 ± 8.21 vs. 7.64 ± 9.45), corresponding to an estimated difference of €716.32 per patient. The adjusted LOS reduction observed in the S-FoCUS-only subgroup (2.40 days) corresponded to an exploratory estimated saving of approximately €1418 per patient (Table 3).

### 3.3. Diagnostic Performance

The distribution of predefined cardiac abnormalities recorded during each study period is summarized in Table 4. The frequency of reported findings was similar between the two periods but complex aortic plaques were more frequently reported in the post-S-FoCUS period. Among patients who underwent both S-FoCUS and TTE during admission (*n* = 62), inter-modality agreement for the detection of predefined high-risk cardiac abnormalities was high, as shown in Table 5. Of 29 patients with unremarkable S-FoCUS, only two (6.9%) had a potentially relevant abnormality according to TTE (one left ventricular hypokinesia, and one severe left atrial enlargement). On the other hand, of the 76 patients with lacunar, atherothrombotic or rare-cause stroke, 11 (14.5%) had clinically significant findings that might have been missed in a setting where cardiac ultrasound is performed only in patients with ESUS or suspected cardioembolism. Raw 2 × 2 agreement data and case examples are provided in Supplementary Table S6 and Table S7, respectively.

## 4. Discussion

We present the results of implementing a neurologist-led S-FoCUS protocol in the acute stroke care pathway in clinical practice. Our data show that S-FoCUS expedites the cardiac ultrasound pathway and halves the number of TTE examinations, highlighting its potential impact on workflow efficiency and resource utilization. Our results indicate that S-FoCUS serves as a front-end triage tool that enables early bedside cardiac assessment while reserving comprehensive echocardiography for specific patients. The screening did not cause any delays in TTE or prolong the hospital stay for patients who required additional examinations. Furthermore, S-FoCUS was linked to a decrease in the hospital LOS in patients with suspected ischemic stroke or TIA. Consequently, S-FoCUS should be regarded as a triage strategy that optimizes early cardiac assessment in the presence of diagnostic uncertainty, rather than a substitute for TTE.

Although evidence within stroke systems remains limited and neither European nor American stroke guidelines currently recommend routine S-FoCUS in the standard workup for stroke diagnosis, earlier experience indicates that neurologist-led focused cardiac ultrasound programs are feasible and can detect clinically relevant cardiac disease. This supports the selective integration of S-FoCUS within stroke networks [18]. In parallel, European cardiovascular imaging societies are developing FoCUS training frameworks and scopes of practice [11].

Our findings provide additional evidence that point-of-care ultrasound programs can streamline inpatient workflows by reducing formal echocardiography requests, imaging burden, and hospitalization costs when integrated into medical services [19]. In fact, FoCUS is most effective for diagnosis when directed at well-defined clinical questions [20].

Contemporary stroke guidelines advocate for the selective, management-oriented use of comprehensive echocardiography [6]. In this context, S-FoCUS serves as a triage tool that allows neurologists to directly visualize high-risk abnormalities, such as severe atrial enlargement or ventricular dysfunction, which warrant formal quantification. This transition to an imaging-based selection process ensures that specialized cardiology resources are reserved for high-yield cases. The agreement observed for predefined high-risk cardiac abnormalities supports the feasibility of using S-FoCUS as an initial triage tool and suggests that it can facilitate earlier cardiac assessment while preserving access to comprehensive TTE for selected cases, as previously reported [12]. Integrating S-FoCUS into the acute stroke pathway could help reduce workload burden and logistical delays by enabling trained neurologists to perform initial cardiac imaging. From a health economic standpoint, our pathway aligns with observational data indicating that selective strategies outperform echocardiography in unselected stroke populations. A recent cost-effectiveness analysis of patients with ischemic stroke or TIA found that universal TTE performance yielded minimal health gains at substantially higher costs. In contrast, selective use based on simple clinical filters was markedly more cost-effective [21,22]. Real-world data also demonstrate significant variation between centers and potential overuse of echocardiography in acute stroke workflows. This underscores the need for triage tools that standardize referrals and focus comprehensive studies where they are most beneficial [23]. These organizational benefits align with the Action Plan for Stroke in Europe 2018–2030, which emphasizes pathway organization, equitable access, and the efficient use of resources across the stroke care continuum [24].

However, it is important to note that S-FoCUS does not replace comprehensive echocardiography or transesophageal echocardiography for certain conditions. It should be used to answer specific questions at the bedside. Notably, S-FoCUS does not visualize the left atrial appendage, provides only a qualitative assessment of chamber size and systolic function, and does not offer formal quantification of valve disease or gradients. Therefore, escalation to comprehensive TTE/TEE is warranted in cases of suspected patent foramen ovale, atrial thrombus, left ventricular dysfunction, valvular pathology, or intracardiac masses, nodules, or vegetations, as these findings may orient the differential diagnosis but still require standard imaging for definitive interpretation [25,26].

This study has limitations. First, the agreement analysis may be subject to confirmation bias since comprehensive TTE was requested only in patients with abnormal or uncertain S-FoCUS findings. Thus, cardiologists were not blinded to the indication. False-negative S-FoCUS results were identified within the subgroup that underwent both tests (Supplementary Table S6). However, because most patients with apparently normal S-FoCUS did not undergo confirmatory TTE, the present study cannot provide a robust estimate of the false-negative rate among negative S-FoCUS examinations. Moreover, blinded validation of S-FoCUS performance had already been conducted previously [16]. Therefore, the results should not be interpreted as demonstrating diagnostic equivalence between S-FoCUS and TTE, nor as excluding missed abnormalities after a negative S-FoCUS examination, but rather as supporting the feasibility of a triage strategy within the diagnostic pathway. Second, the post-implementation cohort is subject to potential selection bias because allocation to S-FoCUS versus direct TTE was pragmatic rather than protocolized. While some patients underwent direct TTE because of known complex cardiac disease requiring comprehensive assessment, others did so for operational reasons such as operator workload, temporary unavailability, or physician request. Accordingly, subgroup comparisons within the post-implementation period, especially with respect to length of stay, should be interpreted cautiously. Moreover, stroke mimics were included in the main cohort because the pathway is activated on the basis of initial clinical suspicion of stroke/TIA, before definitive diagnostic confirmation, which may be delayed in our setting when brain MRI is not immediately available. In sensitivity analyses restricted to confirmed ischemic stroke/TIA, the reductions in time to first cardiac ultrasound assessment and in standard TTE use remained consistent, whereas the difference in length of stay was attenuated and lost statistical significance in the overall post-implementation cohort. Although a shorter length of stay remained evident in the subgroup initially screened with S-FoCUS, this observation should also be interpreted with caution given the pragmatic, non-randomized allocation. Third, our economic analysis was simplified and exploratory. It was based on institutional per diem accounting estimates and was intended only to provide an approximate hospital-based estimate of the potential economic impact associated with differences in LOS. It did not constitute a formal cost-effectiveness analysis and did not capture all relevant components of resource use, including downstream testing, post-discharge costs, or clinical outcomes. Therefore, the economic findings should be interpreted cautiously and confirmed in prospective studies with a formal health economic methodology. Bedside S-FoCUS may also offer workflow advantages by avoiding patient transport for initial cardiac assessment, although these organizational effects were not formally measured in the present study. Finally, generalizability is limited because all S-FoCUS examinations in this study were performed by a single board-certified vascular neurologist with extensive prior experience in neurosonology and supervised FoCUS training. Therefore, the observed workflow benefits and agreement rates may reflect a favorable implementation setting and should not be directly extrapolated to junior neurologists or to centers without similar expertise. Although structured training frameworks are available and recent multicenter validation studies support the feasibility of neurologist-performed FoCUS under supervised conditions [16], broader implementation will require local training, quality assessment, and periodic competency review. Future work should validate these findings in multicenter, pragmatic settings and include formal economic evaluations. Additionally, S-FoCUS should be integrated within standardized training and quality assurance frameworks in more centers.

## 5. Conclusions

Our findings support the feasibility of S-FoCUS as an efficient strategy to expedite cardiac assessment without delaying TTE in our clinical setting; however, they should be interpreted in the context of a single-center experience, and confirmation in broader healthcare settings would help to establish their generalizability.

## Figures and Tables

**Table 1:** Baseline characteristics by study period.

	Pre-S-FoCUS (*n* = 224)	Post-S-FoCUS (*n* = 229)	*p*-Value
Age, median (IQR), years	77 (64–85)	75 (62–83)	0.146
Female sex, *n* (%)	104 (46)	94 (41)	0.248
NIHSS on admission, median (IQR)	3 (1–9)	4 (1–9)	0.327
Final diagnosis, *n* (%)			0.451
Ischemic stroke	181 (81)	187 (82)	
TIA	20 (9)	14 (6)	
Stroke mimics	23 (10)	28 (12)	

**Table 2:** Efficiency outcomes by study period.

	Pre-S-FoCUS (*n* = 224)	Post-S-FoCUS (*n* = 229)	*p*-Value	S-FoCUS * (*n* = 180)	*p*-Value
Time to first cardiac ultrasound assessment , days, median (IQR)	3 (2–5)	1 (1–2)	*p* < 0.001	1 (1–2)	*p* < 0.001
Imaged within 24 h, *n* (%)	12 (5.4%)	143 (63.8%)	*p* < 0.001	129 (71.7%)	*p* < 0.001
Length of stay, days, median (IQR)	6 (4–9)	5 (3–10)	*p* = 0.014	5 (3–9)	*p* < 0.001

* Patients undergoing S-FoCUS screening.

**Table 3:** Multivariable analysis of length of stay adjusted for admission NIHSS.

Comparison vs. Pre-S-FoCUS	Adjusted Difference in LOS, Days	95% CI	*p*-Value	Estimated Savings per Patient
Post-S-FoCUS overall	−0.74	−2.35 to 0.87	0.367	€438
Post-S-FoCUS ± TTE	−1.15	−2.86 to 0.56	0.187	€680
Post direct TTE only	+0.78	−2.01 to 3.58	0.582	−€464
Post S-FoCUS + TTE	+1.22	−2.41 to 4.85	0.509	−€723
Post S-FoCUS only	−2.40	−3.81 to −0.99	0.001	€1418

**Table 4:** Echocardiographic findings in the study periods.

Finding, *n* (%)	Pre-S-FoCUS (*n* = 224)	Post-S-FoCUS (*n* = 229)	*p*-Value
Left atrial enlargement	105 (46.9)	111 (48.5)	0.734
Mitral annular calcification	27 (12.1)	16 (7.0)	0.066
Mitral stenosis	7 (3.1)	12 (5.2)	0.262
Left ventricular dilation	3 (1.3)	10 (4.4)	0.054
Depressed LVEF	13 (5.8)	21 (9.2)	0.174
LV wall-motion abnormality	22 (9.8)	21 (9.2)	0.813
Severe LV hypertrophy	10 (4.5)	8 (3.5)	0.597
Aortic stenosis	13 (5.8)	10 (4.4)	0.486
Dilated aortic root	5 (2.2)	6 (2.6)	0.789
Complex aortic plaques	0 (0.0)	9 (3.9)	0.003
Masses, thrombi, vegetations	4 (1.8)	1 (0.4)	0.169

Findings according to standard TTE when available or S-FoCUS for patients without TTE.

**Table 5:** Concordance between S-FoCUS and standard TTE findings.

*n* = 62	S-FoCUS *n*, (%)	Standard TTE *n*, (%)	Kappa Index	95% CI (Bootstrap)
Depressed LVEF (<50%)	10 (16.1)	10 (16.1)	0.880	0.681–1
Mitral Stenosis	6 (9.7)	4 (6.5)	0.783	0.384–1
Left Ventricular Dilation or Apical Aneurysm	10 (16.1)	6 (9.7)	0.716	0.413–0.938
Severe Left Atrial Dilation	9 (14.5)	9 (14.5)	0.742	0.402–0.943
Left Ventricular Hypo/akinesia	12 (19.4)	11 (17.7)	0.839	0.626–1
Mitral Calcification	7 (11.3)	6 (9.7)	0.914	0.659–1
Aortic Stenosis	5 (8.1)	5 (8.1)	1	1–1
Severe Mitral Regurgitation	3 (4.8)	5 (8.1)	0.734	0.328–1
Dilated Aortic Root	4 (6.5)	4 (6.5)	0.732	0.243–1
Complex aortic plaques	2 (3.2)	1 (1.6)	0.663	0.172–1
Masses, thrombi, vegetations	0 (0.0)	0 (0.0)	–	–

## Data Availability

The data presented in this study are available on reasonable request from the corresponding author. The data are not publicly available due to institutional privacy regulations.

## Supplementary Materials

The following supporting information can be downloaded at: https://www.mdpi.com/article/10.3390/diagnostics16101491/s1, Table S1. Reason for direct TTE without prior S-FoCUS. Table S2. Retrospective reasons for escalation from S-FoCUS to standard TTE in the post-implementation cohort (*n* = 62). Table S3. Sensitivity analysis restricted to patients with ischemic stroke or TIA. Table S4. Sensitivity analysis restricted to patients with confirmed ischemic stroke. Table S5. Sensitivity analysis restricted to patients without indications for direct TTE (ie cardiology consultation, prosthetic valves, suspicion of infective endocarditis or NBTE). Table S6. Raw 2 × 2 agreement data between S-FoCUS and comprehensive TTE in patients who underwent both tests during admission (*n* = 62). Table S7. Summary of cases with no previous suspicion of cardioembolic etiology and abnormal S-FOCUS with clinically relevant implications.
