# Supplementary material for: Efficiency of Neurologist-Led Focused Cardiac Ultrasound in the Acute Stroke Pathway (S-FoCUS)
Source: Diagnostics (Basel). 2026 May 14;16(10):1491. doi: 10.3390/diagnostics16101491 (PMC13205252; doi:10.3390/diagnostics16101491)
Supplement: Supplementary file 1 [file diagnostics-16-01491-s001.zip › diagnostics-4235129-supplementary.pdf]

Supplementary Table S1. Reason for direct TTE without prior S-FoCUS

	n (%)
No specific reason recorded	17 (34.7)
S-FoCUS unavailable	6 (12.2)
Anticoagulation failure	4 (8.2)
Cardiology indication, arrhythmia, or other cardiac issue	4 (8.2)
Mechanical prosthetic valve	4 (8.2)
Known mitral stenosis/prolapse or previous mitral valve surgery	3 (6.1)
Suspected infective endocarditis	3 (6.1)
Cancer / suspected non-bacterial thrombotic endocarditis	2 (4.1)
Poor acoustic window	1 (2.0)
Patient intolerance to bedside S-FoCUS in the acute phase	1 (2.0)
Young patient	1 (2.0)
Patent foramen ovale	1 (2.0)
Early recurrent ESUS	1 (2.0)
Aortic dissection	1 (2.0)

Supplementary Table S2. Retrospective reasons for escalation from S-FoCUS to standard TTE in the post-implementation cohort (n = 62).

	n (%)
No specific reason recorded	28 (45.2)
Cardiology indication (arrhythmia/other)	8 (12.9)
Patent foramen ovale	6 (9.7)
Young patient	5 (8.1)
Anticoagulation failure	4 (6.5)
New-onset atrial fibrillation	4 (6.5)
Mechanical prosthetic valve	2 (3.2)
Suspected infective endocarditis	2 (3.2)
Cancer / suspected NBTE	2 (3.2)
Poor acoustic window	1 (1.6)

Supplementary Table S3. Sensitivity analysis restricted to patients with ischemic stroke or TIA

Outcome	Pre-S-FoCUS (n = 201)	Post-S-FoCUS (n = 201)	p-value	S-FoCUS* (n = 156)	p-value
Time to first cardiac imaging, days, median (IQR)	3 (2–5)	1 (1–2)	<0.001	1 (1–2)	<0.001
Imaged within 24 h, n (%)	10 (5.0)	127 (63.2)	<0.001	110 (70.5)	<0.001
Length of stay, days, median (IQR)	6 (5–9)	6 (4–11)	0.063	5 (4–10)	0.007
TTE performed, n (%)	201 (100)	100 (49.8)	<0.001	55 (35.3)	<0.001

Supplementary Table S4. Sensitivity analysis restricted to patients with confirmed ischemic stroke

Outcome	Pre-S-FoCUS (n = 181)	Post-S-FoCUS (n = 187)	p-value	S-FoCUS* (n = 144)	p-value
Time to first cardiac imaging, days, median (IQR)	3 (3–5)	1 (1–2)	<0.001	1 (0–2)	<0.001
Imaged within 24 h, n (%)	7 (3.9)	118 (63.1)	<0.001	102 (70.8)	<0.001
Length of stay, days, median (IQR)	7 (5–10)	6 (4–11)	0.048	5 (4–10)	0.003
TTE performed, n (%)	181 (100)	95 (50.8)	<0.001	52 (36.1)	<0.001

Supplementary Table S5. Sensitivity analysis restricted to patients without indications for direct TTE (ie cardiology consultation, prosthetic valves, suspicion of infective endocarditis or NBTE)*

Outcome	Pre-S-FoCUS (n = 207)	Post-S-FoCUS (n = 205)	p-value	S-FoCUS* (n = 170)	p-value
Time to first cardiac imaging, days, median (IQR)	3 (3–5)	1 (1–2)	<0.001	1 (1–2)	<0.001
Length of stay, days, median (IQR)	6 (5–9)	5 (3–10)	0.013	5 (3–9)	0.001

*Nonbacterial thrombotic endocarditis

Supplementary Table S6. Raw 2×2 agreement data between S-FoCUS and comprehensive TTE in patients who underwent both tests during admission (n = 62).

Finding	Concordant positive, n	Concordant negative, n	False positive, n*	False negative, n**
Depressed LVEF (<50%)	9	51	1	1
Mitral stenosis	4	56	2	0
Left ventricular dilation or apical aneurysm	6	52	4	0
Severe left atrial dilation	7	51	2	2
Left ventricular hypo/akinesia	10	49	2	1
Mitral calcification	6	55	1	0
Aortic stenosis	5	57	0	0
Severe mitral regurgitation	3	57	0	2
Dilated aortic root	3	57	1	1

Supplementary Table S7. Summary of cases with no previous suspicion of cardioembolic etiology and abnormal S-FOCUS with clinically relevant implications.

Case ID	Stroke etiology	S-FOCUS Finding	Confirmed by TTE
216	Atherothrombotic	Aortic stenosis	Yes, requiring pre-endarterectomy evaluation
13	Undetermined	Severe LVH	Yes, cardiac amyloidosis
49	Rare (hypercoagulability)	LV dilation + depressed LVEF	Yes
124	Undetermined	Mitral stenosis	Yes
134	Undetermined	Depressed LVEF, akinesia	Yes
207	Lacunar	Depressed LVEF	Yes, silent MI requiring stenting
347	Undetermined	Aortic stenosis + LVH	Yes, severe aortic stenosis
351	Lacunar	Depressed LVEF, akinesia, apical aneurysm, aortic stenosis, mitral stenosis	Yes (aortic and mitral stenosis were nonsignificant)

385	Rare (carotid dissection)	Severe mitral regurgitation, severe LA dilation, severe RA dilation	Yes + mitral prolapse and pulmonary hypertension
43	Lacunar	Aortic stenosis, mitral stenosis	Yes
562	Rare (carotid dissection)	Depressed LVEF, akinesia	Yes, dilated cardiomyopathy
